# Efficient and Stable Magnetic Chitosan-Lipase B from *Candida Antarctica* Bioconjugates in the Enzymatic Kinetic Resolution of Racemic Heteroarylethanols

**DOI:** 10.3390/molecules25020350

**Published:** 2020-01-15

**Authors:** Cristina Georgiana Spelmezan, László Csaba Bencze, Gabriel Katona, Florin Dan Irimie, Csaba Paizs, Monica Ioana Toșa

**Affiliations:** Biocatalysis and Biotransformation Research Center, Babeș-Bolyai University, Arany János 11, Cluj-Napoca 400028, Romania; scristina@chem.ubbcluj.ro (C.G.S.); cslbencze@chem.ubbcluj.ro (L.C.B.); gabik@chem.ubbcluj.ro (G.K.); irimie@chem.ubbcluj.ro (F.D.I.); paizs@chem.ubbcluj.ro (C.P.)

**Keywords:** lipase B from *Candida antarctica*, magnetic nanoparticle, chitosan, covalent immobilization, enzymatic kinetic resolution

## Abstract

Lipase B from *Candida antarctica* immobilized by covalent binding on sebacoyl-activated chitosan-coated magnetic nanoparticles proved to be an efficient biocatalyst (49.2–50% conversion in 3–16 h and >96% enantiomeric excess) for the enzymatic kinetic resolution of some racemic heteroarylethanols through transesterification with vinyl acetate. Under optimal conditions (vinyl acetate, *n*-hexane, 45 °C), the biocatalyst remains active after 10 cycles.

## 1. Introduction

In the last decades enzymes have become popular catalysts, not only in academic research, but also in fine chemical production, lipases being by far the most popular enzymes for industrial applications [1]. Alongside their high chemo-, region- and stereoselectivity, lipases are able to transform efficient water-insoluble substrates, since their natural substrates- the triglycerides are also insoluble in water and are transformed at the oil-water interface. The high enantioselectivity shown in the kinetic and dynamic kinetic resolution of racemates turned lipases into powerful tools for the preparation of the enantiomers of pharmaceuticals and fine chemicals [2,3].

In industrial applications not only the catalytic activity, but also the catalyst operational stability is important, thus besides the modulation of enzyme activity and selectivity, the need to increase the stability of the enzymes and to transform them into robust, recyclable catalyst, operating during several catalytic cycles, has become crucial. Accordingly, many effective immobilization methods have been developed, using a large palette of materials as support [4].

Lipase B from *Candida antarctica* (CaL-B), first isolated from the sediments of Lake Vanda in Antarctica [5] possessing a high lipolytic activity [6], was intensively studied, important biomedical application and structural properties [7,8,9] being reported. Based on its high selectivity, commercial availability, high tolerance to organic solvents, temperature and large substrate tolerance, CaL-B is currently used in many industrial applications [1,2,10] and presents a high potential as catalyst in the kinetic resolution processes of secondary chiral alcohols [11], allowing the preparation of both stereoisomers in their enantiopure form.

While enzymatic kinetic resolutions of racemic 1-heteroarylethanols mediated by lipases have been already reported [12,13,14,15,16,17,18,19], herein in our aim to develop robust biocatalysts for process scale-up, immobilization of CaL-B through covalent binding is approached, to obtain stable, storable, easy to handle and as consequence recyclable immobilized lipase preparations, required for preparative scale processes [20,21]. Among the available support materials, nanosupports present several advantages, the immobilized enzymes are available on a large surface diffusion barrier-free, the suspension is free from sedimentation and approximates the behavior of a homogeneous liquid, but their separation is not simple. Graphene, gold, silica, chitosan, diamond, zirconium or magnetic nanoparticles (MNP) were reported as suitable nanosupports [22].The use of MNP supports, besides the above advantages, provides facile separation of the immobilized biocatalysts [23], while for covalent enzyme immobilization, coated MNPs bearing various functional groups have been developed [24]. Exploiting their advantages the magnetic nanoparticles are already used in several chemical and biomedical applications (catalysis, magnetic hyperthermia, bio-separation, biomolecules immobilization and drug delivery), since their facile recovery, recyclability, high mechanical strength, effective enzyme load, mass transfer resistance allows one to reduce the overall costs of the processes [21,22].

During the immobilization procedure the preservation of the active conformation, responsible for enzyme activity and selectivity, and the increased strength of the enzyme-support linkage, responsible for operational stability/recyclability, which strongly depends on selected immobilization procedure [25,26,27]. Usually, enzyme immobilization onto magnetic nanoparticles can be obtained by three methods: physical adsorption, entrapment and covalent attachement [4,28,29,30,31]. Various enzymes such as lipase [32], α-amylase [33], laccase [34], β-D-galactosidase [35] or alcohol dehydrogenase [36] were immobilized onto MNP via physical adsorption, but the obtained enzyme preparations were characterized by reduced reusability [37]. When physical entrapment occurs, more stable biocatalysts were obtained, the enzymatic activity was usually maintained, but the performance of these biocatalysts was often affected by both the diffusion barrier and enzyme leakage [4,28,29].

**Scheme 1:**
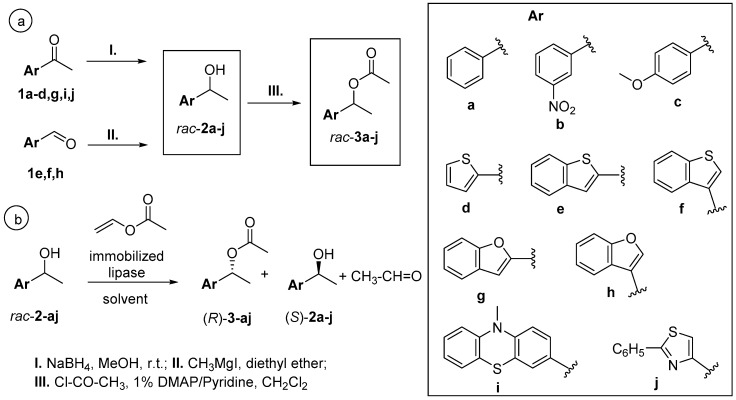
(**a**) Chemical synthesis of racemic compounds (**1**–**2a**–**j**); (**b**) Enantiomer-selective transesterification of racemic ethanols mediated by immobilized CaL-B.

The main objective of this work was to develop efficient EKR processes of various racemic 1-heretoarylethanols (Scheme 1) using novel CaL-B- conjugates as catalyst, assembled through the use of chitosan coated magnetic nanoparticle (MNP-CS) supports, and diverse spacer-arms/linkers for the covalent binding of CaL-B.

Chitosan (CS), a natural oligomer of nine units of glucosamine with varying degrees of deacetylation, was chosen as coating material. Based on its properties such as biocompatibility, biodegradability, hydrophilicity, renewability and the availability of functional groups such as -NH_2_ and -OH, the chemical modifications [38,39,40] of chitosan do not change the fundamental skeleton of the biopolymer and have a beneficial effects on some properties like bacteriostatic effect [32,41,42,43], chelating and adsorption properties. Due to its renewability (is obtained from shells of shellfish: crabs, lobsters, shrimps and krills and also from the waste of the fishing industry), chitosan has a low cost [22] providing competitive prices for the prepared biocatalysts. 

Lipase immobilization can induce conformational changes similar to those resulted by interfacial activation, increasing the catalytic activity. The-NH_2_ functional groups of chitosan provide facile introduction of different spacer-arm between chitosan and biocatalyst and as consequence the immobilization yield can be improved and the activity of covalent immobilized enzyme can be tailored for specific substrate and specific transformations, as result of the interaction of enzyme hydrophobic lid with the spacer arm residues [4]. Common spacer-arms, linkers used in the covalent immobilization of enzymes are: 1-(-3-dimethylaminoproyl)-3-ethylcarbodiimide hydrochloride (EDAC), *N*-hydroxysuccinimide (NHS) [32,44,45,46], glutaradehyde [47], glycerol diglycidyl ether (GDGE) [48,49,50], alkyl diamines [51] and dicarboxylic acid halides [52,53].

## 2. Results and Discussion

### 2.1. Chemical Synthesis of Racemic Heteroaryl-Ethanols rac-2a-i and Their Corresponding Acetates rac-3a–j

Optically pure heteroarylethanols are valuable building blocks for fine chemical synthesis, especially in the pharmaceutical industry [1,2]. The racemic substrates *rac*-**2a**–**d**,**g**,**i**,**j** were obtained through chemical reduction with sodium borohydride of the corresponding prochiral heretoarylmethylketones **1a**–**d**,**g**,**i**,**j**, while the heretoarylethanols *rac*-**2e**,**f**,**h** were obtained by Grignard reaction from the corresponding aldehydes **1e**,**f**,**h**. Further, the racemic ethanols were used as substrates in the enzymatic transesterification reactions and also as starting materials for the synthesis of the corresponding racemic acetates *rac*-**3a**–**j**, used to set-up the chromatographic separation methods (Scheme 1) [16,17,18,54]. 

### 2.2. Covalent Immobilization of CaL-B on MNP-CS Supports

**Scheme 2:**
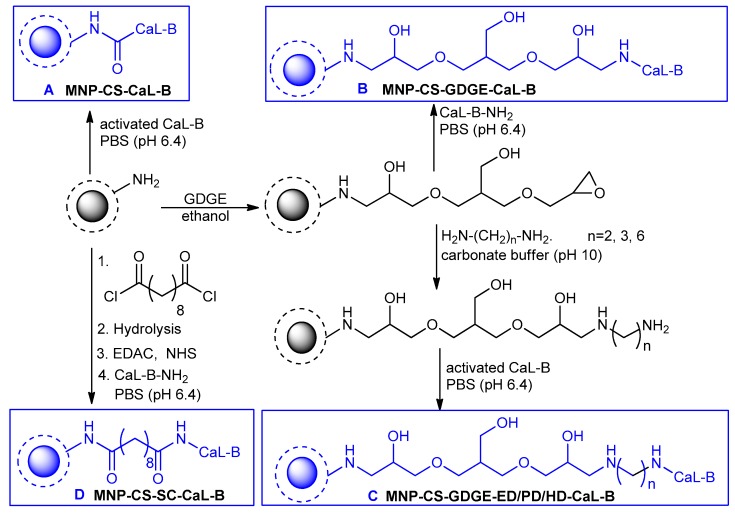
Covalent immobilization of CaL-B on MNP-CS based supports. (**A**) Direct covalent attachment of CaL-B onto MNP-CS; (**B**) Covalent attachment of CaL-B onto MNP-CS using GDGE as linker; (**C**) Covalent attachment of CaL-B onto MNP-CS using GDGE as linker and different diamines as spacer-arms; (**D**) Direct covalent attachment of CaL-B onto sebacoyl-activated MNP-CS.

With the aim to provide an efficient lipase preparate, four approaches were used for the covalent immobilization of CaL-B on chitosan-coated MNP (MNP-CS), involving a variety of interactions between enzyme and the nano-support (Scheme 2): (A) the direct covalent binding of activated enzyme to the chitosan amino groups [32,44,45,46] allows the preparation of the most simple nanobioconjugate, but the newly formed amidic bond can represent an important barrier for lipase mobility since its direct binding to the support generates protein conformational changes, negatively affecting the activity/selectivity; (B) the covalent binding through the residual epoxy groups of glycerol-diglycidyl ether (GDGE) cross-linker, covalently attached to amino groups of chitosan, that allows enzyme binding through a more flexible arm in comparison to the classical glutaraldehyde [48,49,50]; (C) the covalent binding through an alkyl-diamine linker previously bonded to the GDGE activated nanoparticle [51] that provides longer, even more flexible spacer-arm as compared with (B) and (D) covalent binding on the sebacoyl (SC)-derivatized MNP-CS via the activated ester method [52,53], the dicarboxylic acid halides acting as activating group in enzyme immobilization, while the hydrophobic alkyl chain was shown to be beneficial for maintaining the active conformation of the immobilized lipase [53].

Since the lipases’ catalytic activity in organic solvents is not rigorously reflected by their hydrolytic activities determined in aqueous solution, Wang et al. [55] have been developed a colorimetric method based on the amount of released acetaldehyde when activated vinyl esters are used in transesterification reactions, named by them synthetic activity. Using this parameter, a first evaluation was performed. The obtained results for all new biocatalysts, for the lyophilized lipase and Novozym 435 as references are presented in Table 1. 

The synthetic activity of the new enzyme preparation bearing the sebacoyl group was much higher as compared with the lyophilized enzyme (Entries 8 and 1 in Table 1), probably due to the hydrophobic residue reducing the enzyme-enzyme interactions at the support surface. The most active biocatalyst, MNP-CS-SC-CaL-B (Entry 8 in Table 1), has a synthetic activity similar to that of the commercially available Novozym 435 (Entry 2 in Table) when comparing the synthetic activity reported on enzyme preparation weight (column 3 in Table 1). It is notable that despite its widespread use, Novozym 435 is unstable, and even even fragile under stirring, and through breakage of the biocatalyst particles, the enzyme can be released, an important loss of enzyme activity being often observed and reported [56,57], issues which can be alleviated through the use of robust covalent enzyme immobilizates.

### 2.3. Enzyme Catalyzed Transesterification Studies

As known, in the lipase catalyzed transformation of secondary alcohols, the individual enantiomers are transformed with different reaction rates and in accordance to the Kazlauskas-rule the *R*-esters are formed faster, while the slow reacting *S*-ethanols remain untransformed [58]. 

First, in order to confirm the results obtained for the efficiency of the new CaL-B preparations based on the synthetic activity, the new biocatalysts (containing the same quantity of CaL-B) were tested in the EKR of the racemic 1-phenyl-1-ethanol *rac*-**2a** chosen as model substrate, using vinyl acetate as irreversible acyl donor, since the formed vinyl alcohol spontaneous tautomerized to acetaldehyde, favoring the product formation (Scheme 1b).

The conversion *c* and enantiomeric excesses *ee_P_* and *ee_S_* (see Section 3) were used as measure of biocatalyst activity and selectivity, in order to quantify the biocatalyst efficiency.

**Figure 1:**
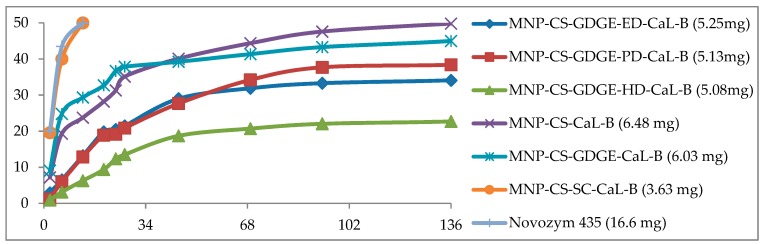
Biocatalyst screening in the EKR of *rac*-**2a**.

The obtained results (Figure 1) are in accordance with the activity study; as consequence MNP-CS-SC-CaL-B was chosen as optimal biocatalyst, allowing the maximum conversion (≈50%) in shorter reaction time (only 13 h) and high enantiomeric excesses (ee_S_, ee_P_ > 99%).

**Figure 2:**
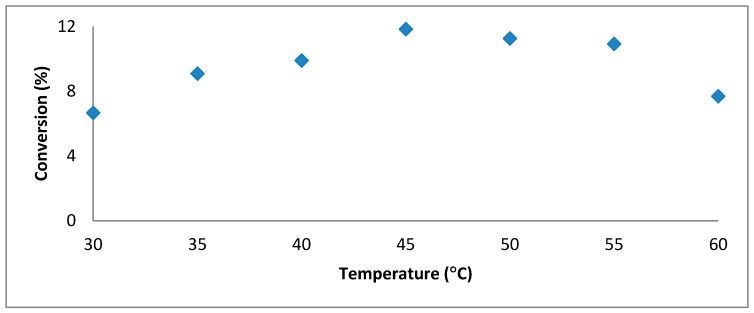
The temperature effect on enzymatic activity of MNP-CS-SC-CaL-B (conversion after two hours).

Since the temperature has an important effect on the reaction rate and based on the known high termostability of CaL-B, the EKR of racemic 1-phenyl-1-ethanol *rac*-**2a** with vinyl acetate in *n*-hexane was performed at temperature gradually increased with 5 °C between 30 and 60 °C. Samples were taken after 2 h and analyzed on HPLC. A continuous increased was observed until 45 °C; afterwards the conversion decreased (Figure 2). The obtained results are in concordance with the literature data, reporting an increased termostability of CaL-B bioconjugates as compared with the free enzyme [59]. As consequence 45 °C was chosen as optimal temperature, all further experiments were performed at this temperature.

The nature of the solvent can play an important role in the enzyme efficiency, since the enzyme conformation strongly depends on the reaction media. Five nonpolar and polar aprotic solvents were used in a similar manner for the model substrate. The obtained experimental data confirm as optimal solvent the *n*-hexane (42% conversion after 6 h, as compared with approx. 12% conversion obtained in toluene and under 2% in the more polar tetrahydrofuran, methyl *tert*-butyl ether and dichloromethane).

Next, aiming to enhance the productivity of EKR process, the effect of substrate-enzyme *ratio* was studied using similar conditions (5 mg of substrate and 2 equiv. of vinyl acetate in *n*-hexane- 1 mL, at 45 °C and 1000 rpm), modifying the quantity of enzyme. Samples were taken after 6 h and analyzed on HPLC. The conversion increases from 12% at 15:1 *ratio* (w*_rac_*_-**2a**_/w_biocatalyst_) as compared to approx. 45% at 5:1 *ratio*, decreasing to 30% when a 2.5:1 *ratio* was used. The optimal substrate- enzyme *ratio* established in conclusion to 5:1 was used in further experiments.

Since the nature of the acyl donor could also significantly influence the selectivity of the enantiomer selective acylation of secondary alcohols catalyzed by CaL-B [60], the acylation of *rac*-**2a** was next performed in the already established optimal conditions (*n*-hexane, at 45 °C, 5:1 *ratio* and 1000 rpm) with three activated vinyl esters: vinyl acetate, butyrate and decanoate (2 equiv.). Vinyl acetate proved to be the best acylation agent, since the maximum conversion (50%) was reached in the shortest reaction time (13 h, Table 2, Entry 1).

**Figure 3:**
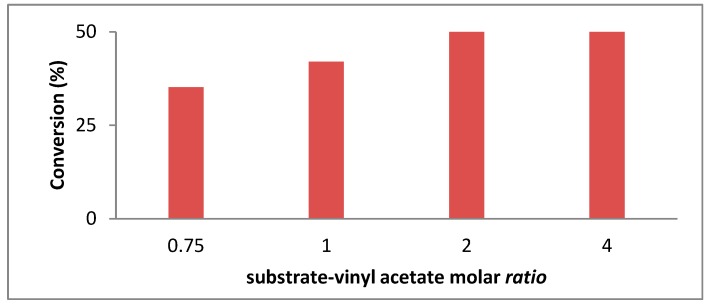
The influence of substrate: vinyl acetate molar *ratio* on the conversion of *rac*-**2a**.

Further the effect of the substrate-vinyl acetate molar *ratio* was investigated using the same amount of *rac*-**2a** (5 mg) and biocatalyst CaL-B (1 mg), in *n*-hexane (1 mL), at 45 °C. Samples were taken after 13 h and analyzed on HLPC. The highest activity (50% conversion after 13 h) and selectivity (*ee_S_, ee_P_* > 99.9%) resulted at 1:2 and 1:4 molar *ratio* with vinyl acetate (Figure 3).

### 2.4. Analytical-Scale Lipase-Mediated O-Acylation of Racemic Heteroarylethanols rac-**2a**–**j**

The substrate domain for the new biocatalyst was investigated next using the determined optimal conditions (Scheme 1b, Table 3). Samples were taken periodically from the reaction mixtures, diluted with *n*-hexane and analyzed on appropriate HPLC chiral columns (Table 3). Very good results (conversions > 49% and high optical purities, *ee_P_* > 99%, *ee_S_* > 96%, *E* > 200) [61], were obtained for all tested substrates, demonstrating the excellent catalytic properties and a large substrate domain of MNP-CS-SC-CaL-B.

### 2.5. Reuse Experiments.

**Figure 4:**
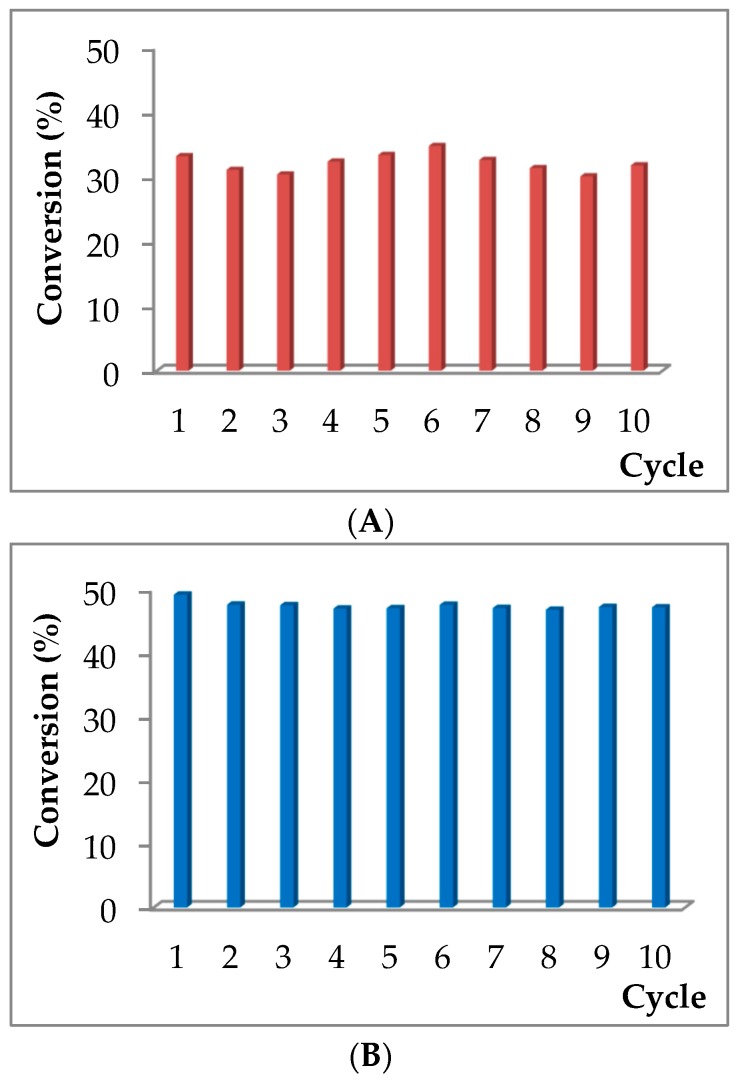
The reusability of the optimal biocatalyst MNP-CS-SC-CaL-B in the EKR of: (**A**). *rac*-**2e** (1 h reaction time) and (**B**). *rac*-**2i** (10 h reaction time).

The reusability of the best performing biocatalyst, MNP-CS-SC-CaL-B, was finally studied in the enantioselective acylation of *rac*-**2e** (Figure 4A) since it represents an important requirement for any application, allowing the development of sustainable processes.

The reaction was performed 10 times with the same biocatalyst as previously described. After each cycle, the enzyme preparate was washed with *n*-hexane (3 × 0.5 mL) and immediately reuse in the next cycle.

The recycling capacity, but also the long term stability of the enzyme preparate was studied similar to the EKR of 1-(10-methyl-10*H*-phenothiazin-3-yl)ethan-1-ol *rac*-**2i** (Figure 4B), taking samples after 10 h in order to rich higher conversions, closed to 50%.

As can be observed in Figure 4A, B the activity remains high in both cases even after 10 cycles, decreasing by less than 5%. As consequence, the operational and long term stability of the new prepared immobilized lipase is promising for further experiments in a continuous-flow system, allowing higher productivity.

## 3. Materials and Methods 

Lipase B from *Candida antarctica* (CaL-B) was purchased as solution from Novozymes (Copenhagen, Denmark). Medium molecular weight chitosan (50–190 kDa, 75–85% deacetylated), iron chloride (FeCl_2_ and FeCl_3_) were purchsed from Sigma-Aldrich (Darmstadt, Germany). Sebacoyl chloride, glycerol diglycidyl ether (GDGE), ethylenediamine (ED), 1,3-diaminopropane (PD), 1,6-diaminohexane (HD), 1-(-3-dimethylaminopropyl)-3-ethylcarbodiimide hydrochloride (EDAC), *N*-hydroxysuccinimide (NHS), 3-methyl-2-benzothalininone hydrazone hydrochloride (MBTH), ammonium iron(III) sulfate, vinyl acetate and all solvents used in the enzymatic reactions with highest purity were purchased from Sigma-Aldrich, Merck (Darmstadt, Germany), Alfa Aesar (Kandel, Germany) or VWR Chemicals (Darmstadt, Germany).

Solvents used in the chemical synthesis were used as purchased (methanol, diethyl ether) or dried over molecular sieve (dichloromethane). 

Chiral HPLC (Agilent 1200 Series instrument, Agilent, Waldbronn, Germany) was used for the quantitative analysis of the enzymatic kinetic resolution products, samples being taken periodically and investigated by chiral chromatography.

The enantiomeric excesses of the substrate and of the product (*ee_S_*, *ee_P_*) were calculated from peak areas of HPLC chromatograms. The enantiomeric *ratio* (*E*) was calculated with the known Equation (1) using the conversion *c* calculated with the equation (2) as well-known [61]:

(1) $$E=\frac{ln\left[\left(1-c\right)\times \left(1-ee_{S}\right)\right]}{ln\left[\left(1-c\right)\times \left(1+ee_{S}\right)\right]}$$

(2) $$c=\frac{ee_{S}}{\left(ee_{S}-ee_{P}\right)}$$

For the quantitative spectrophotometric determinations of enzyme load through BCA method, an Agilent 8453 UV-Vis spectrophotometer equipped with thermostat was used. An Alpha 1-2 LO plus lyophilizer (Gore, Felkirchen-Westerham, Germany) was used to dry the intermediate materials and newly obtained enzyme preparations. 

Thin layer chromatography (TLC) was performed on 0.2 mm Kieselgel sheets (Macherey-Nagel, Merck, Darmstadt, Germany) with UV-254 fluorescent indicator. Spots were visualized by treatment with 5% ethanolic phosphomolibdic acid, vanillin or 2,4-dinitrophenylhydrazine solution (depending on the investigated compound), followed by heating. For all the experiments a Heidolph Vibramax 1000 shaker, equipped with an incubator module (Heidolph, Schwabach, Germany) was used.

### 3.1. Chemical Synthesis

The synthesis of the investigated racemic substrates *rac*-**2a**–**j** and products *rac*-**3a**–**j** was performed using known methods [16,17,18,54]. The racemic heteroarylethanols were obtained from the corresponding heteroarylmethylketones (compounds **1a**–**d**,**g**,**i**,**j**) by chemical reduction with sodium borohydride or from the corresponding aldehydes (compounds **1e**,**f**,**h**) by Grignard reaction. Next, the racemic ethanols (*rac*-**2a**–**j**) were transformed into the corresponding acetates (*rac*-**3a**–**j**) using acetyl chloride (1.5 equiv.) and a catalytic amount of 1% DMAP/pyridine in dry CH_2_Cl_2_ (Scheme 1a).

### 3.2. Immobilization of Lipase B from Candida Antarctica (CaL-B) on Magnetic Nanoparticles Coated with Chitosan (MNP-CS)

#### 3.2.1. Synthesis of MNP-CS

Magnetic nanoparticles coated with chitosan (MNP-CS) were prepared through co-precipitation as described earlier [27,32]. Chitosan (2 g) was suspended in acetic acid solution (1%, 200 mL) and kept under magnetic stirring (600 rpm) for 1 h, until complete dissolution occurs. FeCl_2_ (7.4 g, 0.05 mol) and FeCl_3_ (20.2 g, 0.12 mol) were added into the prepared solution and left under stirring for 30 min. A solution of NaOH (30 %, 70 mL) was added dropwise, resulting in a black precipitate, the magnetic nanoparticles coated with chitosan (MNP-CS) which were magnetic separated, washed with distilled water (5 × 10 mL) and vacuum freeze dried. 

#### 3.2.2. Covalent Immobilization of CaL-B on MNP-CS

(A) For the direct covalent attachment of CaL-B onto MNP-CS the enzyme was first activated with EDAC. Into the solution of CaL-B (2 mL, 8.3 mg/mL) diluted with saline phosphate buffered (PBS, 50 mM, pH 6.4, 1 mL) 1 mg of EDAC dissolved in PBS (50 mM, pH 6.4, 50 µL) was added. The mixture was stirred (600 rpm) at room temperature for 1 h. The obtained solution was added into the suspension of MNP-CS (50 mg) in PBS (50 mM, pH 6.4, 2 mL) and the resulted suspension was left under stirring (600 rpm) at room temperature for 2 h. The obtained biocatalyst (Scheme 2A) was separated with a magnet, washed with distilled water (3 × 0.5 mL) and vacuum freeze dried [44,45,46].

(B) The second strategy is based on the use of GDGE as linker. Over the MNP-CS (50 mg) suspended in absolute ethanol (5 mL) GDGE (250 µL) was added and the mixture was left under stirring at room temperature for 8 h. The resulted support (MNP-CS-GDGE) was separated by a magnet and washed with distilled water (3 × 0.5 mL). Finally the CaL-B solution (2 mL, 8.3 mg/mL) was added over the previously obtained MNP-CS-GDGE support suspended in PBS (50 mM, pH 6.4, 2 mL) and left under stirring at room temperature overnight. The new biocatalyst was separated with a magnet, washed with distilled water (3 × 0.5 mL) and vacuum freeze dried (Scheme 2B) [48,49,50].

(C) In the third strategy diamines of different length were used as spacer-arms between the support (GDGE-derivatized chitosan-coated MNP) and lipase. MNP-CS-GDGE (50 mg) were suspended in carbonate buffer (50 mM, pH 10, 2 mL) and left under stirring for 1 h, when ethylenediamine, 1,3-diaminopropane or 1,6-diaminohexane (ED, PD or HD, 600 mg in each case) was added and the reaction was further performed at room temperature under stirring for another 2 h. The three new derivatized supports (MNP-CS-GDGE-ED/PD/HD) were separated using a magnet and washed with distilled water (3 × 2 mL). To each support (MNP-CS-GDGE-ED/PD/HD) suspended in PBS (50 mM, pH 6.4, 2 mL) a solution of EDAC activated CaL-B (2 mL, 8.8 mg/mL, see section A) was added and left under stirring at room temperature overnight. The new biocatalysts were separated with a magnet, washed with distilled water (3 × 0.5 mL) and vacuum freeze dried. (Scheme 2 C) [51].

(D) The fourth type was obtained after MNP-CS derivatization with sebacoyl chloride (SC) (Scheme 2D). The remained free carboxylic groups generated after hydrolysis were finally conjugated with CaL-B through the active ester method using EDAC and NHS. MNP-CS (50 mg) were suspended in *n*-hexane (2 mL) containing SC (320 µL) and stirred for 10 min. After magnetic separation, the preparation (MNP-CS-SC) was washed with *n*-hexane (3 × 1 mL) for removing the unreacted SC and dried at room temperature. The complete hydrolysis of the unreacted acyl chloride groups was performed immersing the MNP-CS-SC in deionized water at 37 °C for 30 min. The MNP-CS-SC were submerged in saline phosphate buffered (PBS, 50 mM, 100 mM NaCl, pH 6.4, 2 mL) containing EDAC (0.1 mol, 15.5 mg) and NHS (0.1 mol, 11.5 mg) with gentle shaking at room temperature for 1 h. The resulted activated nanoparticles were separated and washed with deionized water (3 × 1 mL). Next, the activated magnetic nanoparticles were immersed in PBS (50 mM, pH 6.4, 2 mL) containing CaL-B (3 mL, 7.47 mg/mL) and left under stirring overnight. In order to completely remove the physically adsorbed enzyme, the biocatalyst was separated and washed with distilled water (5 × 1 mL) and finally vacuum freeze dried for 24 h (Scheme 2D) [52,53].

#### 3.2.3. CaL-B Load on MNP-CS Based Supports

The immobilization yields and the enzyme load were calculated for each case using the protein content of the initial protein solution, of the separated filtrate and of all unified washing solutions, determined spectrophotometrically with the BCA assay (see Table 4).

#### 3.2.4. The Protocol for the Synthetic Activity 

**Scheme 3:**
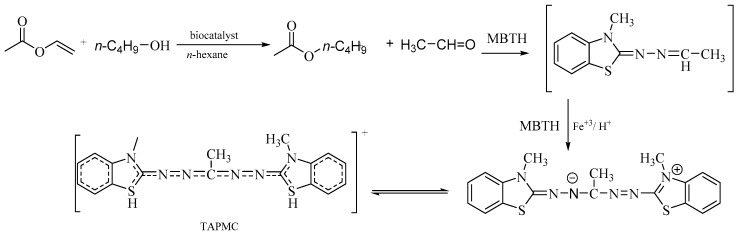
The reaction involved in the synthetic activity quantification.

A fast and sensible colorimetric method was used for the synthetic activity of the new biocatalyst evaluation. The method is based on *n*-butanol transesterification with vinyl acetate (2 equiv.) in *n*-hexane at 30 °C and 1000 rpm. The released acetaldehyde was determined after derivatization with 3-methyl-2-benzothalininone hydrazone (MBTH) as hydrochloride salt, when the formed aldazine is further converted by oxidative coupling with another MBTH molecule in the presence of ammonium iron(III) sulfate (NH_4_Fe(SO_4_)_2_ × 12H_2_O) into a blue tetraazapentamethylene-cyanine (TAPMC) with a maximum absorption at 598 nm (Scheme 3) [55].

The test transesterification reaction using as substrate *n*-butanol (9.14 µL), vinyl acetate (2 Eq.) as acylation agent, 1 mg of enzymatic preparation and *n*-hexane (1 mL) as solvent, was performed at 30 °C for 5 min. A sample of 5 µL was taken from the reaction mixture, mixed with MBTH solution (1% in dH_2_O, 400 µL) and double distilled water (395 µL). The mixture was left for 10 min at 30 °C and H_4_FeNO_4_S_2_ × 12H_2_O solution (1% in HCl 10%, 200 µL) was added; the resulted mixture was stirred for another 30 min at the same temperature and the absorbance at 598 nm was finally measured [55].

#### 3.2.5. General Protocol for Enzymatic *O*-Transesterification

In order to find the best performing biocatalyst, all new enzyme preparates were tested on racemic 1-phenyl-1-ethanol *rac*-**2a** as model substrate. The EKR process was further optimized regarding the solvent, acylation agent, quantity of enzyme, quantity of acylation agent and temperature. The substrate domain in the optimal conditions was finally established.

#### 3.2.6. Enzyme Catalyzed Transesterification Studies

##### Determination of the Optimal Biocatalyst

The new biocatalysts were tested in the EKR of the racemic 1-phenyl-1-ethanol *rac*-**2a** chosen as model substrate (5 mg) with vinyl acetate (2 equiv.) as acylation agent, using *n*-hexane (1 mL) as solvent and the quantity of each biocatalysts containing 1 mg of CaL-B, at 50 °C and 1000 rpm. Samples were taken every 2 h and analyzed on HPLC (Figure 1).

##### Establishing of the Optimal Temperature

The EKR of 1-phenyl-1-ethanol *rac*-**2a** (5 mg) with vinyl acetate (2 equiv.) in *n*-hexane (1 mL) in presence of the biocatalyst (3.63 mg enzyme preparate containing 1 mg of lipase) was next performed at temperature gradually increased with 5 °C between 30 °C and 60 °C at 1000 rpm. Samples were taken after 2 h and analyzed on HPLC (Figure 2).

##### Determination of the Optimal Solvent

The mixtures of the substrate (*rac*-**2a**, 5 mg), vinyl acetate (2 equiv), biocatalyst (3.63 mg enzyme preparation containing 1 mg of lipase) and the tested solvent (1 mL) were shaken (1000 rpm) in reaction vials at the optimal temperature (45 °C). Samples were withdrawn after 6 h, diluted, filtered and analyzed through HPLC.

##### Optimal Substrate-Enzyme *Ratio*

The effect of substrate-enzyme *ratio* was studied using 5 mg of *rac*-**2a** as substrate, 2 equiv. of vinyl acetate in *n*-hexane (1 mL), at 45 °C and 1000 rpm), modifying the quantity of enzyme. Samples were taken after 6 h and analyzed on HPLC. 

##### Optimal Acyl Donor

The acylation of *rac*-**2a** (5 mg) was next performed in the optimal established conditions with three vinyl esters: vinyl acetate, vinyl butyrate and vinyl decanoate (2 equiv.) in *n*-hexane (1 mL) with the optimal biocatalyst (3.63 mg enzyme preparation containing 1 mg of lipase) at 45 °C and 1000 rpm (Table 2).

##### Optimal Molar *Ratio* Substrate-Vinyl Acetate

The acylation of model substrate *rac*-**2a** (5 mg), in *n*-hexane (1 mL), with 3.63 mg of biocatalyst (1 mg of CaL-B), was performed at 45 °C and 1000 rpm modifying the quantity of the vinyl acetate (0.75, 1, 2, and 4 equiv.). Samples were taken after 13 h and analyzed on HLPC (Figure 3).

#### 3.2.7. General Protocol for Recycling Experiments

The reusability and the stability of the optimal enzyme preparation was further tested in reuse experiments in the EKR of *rac*-**2e** and *rac*-**2i**, using 5 mg of substrates, 3.63 mg of MNP-CS-SC-CaL-B (containing 1 mg enzyme), 1 mL of *n*-hexane as solvent, 2 equiv. of vinyl acetate at 45 °C and 1000 rpm. Samples were taken after 1 h for *rac*-**2e** and after 10 h for *rac*-**2i**. After each cycle the enzyme preparation was washed with *n*-hexane (3 × 0.5 mL) and immediately reused in the next cycle (Figure 4A,B).

## 4. Conclusions

In this study, the covalent immobilization of CaL-B on chitosan-coated magnetic nanoparticles through covalent binding was achieved. The best performing enzyme preparation, with a sebacoyl group for support activation and as spacer arm, proved to be active and selective in the EKR of various 1-heteroarylethanols. Moreover, based on its high operational stability the new biocatalyst represents a promising candidate for further continuous flow experiments, in order to achieve higher productivities. 

## Figures and Tables

**Table 1:** The synthetic activity of the new obtained biocatalysts.

Entry	Biocatalyst	Synthetic Activity ^a^	
		(mmol/min g_enzyme preparate_)	(µmol/min mg_CaL-B_)
1	Lyophilized CaL-B	0.44	0.44
2	Novozym 435	1.96	32.53
3	MNP-CS-CaL-B	0.13	0.84
4	MNP-CS-GDGE-CaL-B	0.13	0.78
5	MNP-CS-GDGE-ED-CaL-B	0.21	1.1
6	MNP-CS-GDGE-PD-CaL-B	0.20	1.02
7	MNP-CS-GDGE-HD-CaL-B	0.09	0.45
8	MNP-CS-SC-CaL-B	1.95	7.08

^a^ According to the Lambert-Berr law, where ε = 14,300 [55].

**Table 2:** Determination of the optimal acylation agent.

Entry	Acylation Agent	Time (h)	*ee_S_* (%) ^a^	*ee_P_* (%) ^a^	*c* (%) ^a^
1	Vinyl acetate	13	>99.9	>99.9	50
2	Vinyl butyrate	79	>99.9	>99.9	50
3	Vinyl decanoate	24	77.6	85	43.7

^a^ Calculated as described in Section 3.

**Table 3:** EKR of various heteroarylethanols *rac*-**2a**–**j**.

Entry	Substrate	Chromatographic Chiral Separation		Original Results ^b^		Literature Data ^c^	
		Column	*n*-Hexane:IPA	*c ^a^* (%)/Reaction Time (h)	*ee_S_* (%)	*C* (%)/Reaction Time (h)	Ref.
1	*rac*-2a	LUX-3	98:2	50/13	>99.9	37/3	19
2	*rac*-2b	AS-H	95:5-15 min; 85:15	50/5	>99.9	-	-
3	*rac*-2c	LUX-3	95:5	49.7/5	98.9	48/3	19
4	*rac*-2d	LUX-3	95:5	50/4.5	>99.9	49/3	19
5	*rac*-2e	LUX-5	95:5	49.4/4	97.6	49.8^d^/24	17
6	*rac*-2f	LUX-3	99:1-10 min; 90:10	49.2/16	96.9	42.2/24	17
7	*rac*-2g	LUX-3	90:10	49/8	96.1	49/8	16
8	*rac*-2h	LUX-3	90:10	49.6/12	98.2	48/16	17
9	*rac*-2i	LUX-3	70:30	49.2/10	96.8	49^e^/24	18
10	*rac*-2j	LUX-3	90:10	50/3	>99.9	51/5	21

^a^*ee_P_* >99% in all cases, *E* > 200 (calculated as described in Section 4); ^b^ calculated as described in Section 3, with MNP-CS-SC-CaL-B as catalyst; ^c^ with Novozym 435; ^d^ non-selective process; ^e^ with vinyl butanoate.

**Table 4:** The enzyme load in the prepared biocatalyst (Scheme 2).

Entry	Method	Biocatalyst	Enzyme Load (mg_enz_/mg_enzyme preparate_)	Immobilization Yield (%)
1	A	MNP-CS-CaL-B	0.15	54.90
2	B	MNP-CS-GDGE-CaL-B	0.16	59.79
3	C, n=2	MNP-CS-GDGE-ED-CaL-B	0.19	66.88
4	C, n=3	MNP-CS-GDGE-PD-CaL-B	0.19	68.86
5	C, n=6	MNP-CS-GDGE-HD-CaL-B	0.19	69.75
6	D	MNP-CS-SC-CaL-B	0.27	95.96
